# Development of a Paper-Based Sensor Compatible with a Mobile Phone for the Detection of Common Iron Formulas Used in Fortified Foods within Resource-Limited Settings

**DOI:** 10.3390/nu11071673

**Published:** 2019-07-21

**Authors:** Anna W. Waller, Marco Toc, Dylan J. Rigsby, Marcela Gaytán-Martínez, Juan E. Andrade

**Affiliations:** 1 Department of Food Science and Human Nutrition, University of Illinois at Urbana-Champaign, Urbana, IL 61801, USA; 2 School of Art and Design, Department of Industrial Design, University of Illinois at Urbana-Champaign, Champaign, IL 61820, USA; 3 Programa de Posgrado en Alimentos del Centro de la República (PROPAC), Research and Graduate Studies in Food Science, School of Chemistry, Universidad Autónoma de Querétaro, Centro Universitario Cerro de las Campanas s/n Col. Centro, Querétaro 76000, Mexico; 4 Division of Nutritional Sciences, University of Illinois at Urbana-Champaign, Urbana, IL 61801, USA

**Keywords:** iron, paper-based assay, sensor, fortification, mobile app

## Abstract

A lack of quality control tools limits the enforcement of fortification policies. In alignment with the World Health Organization’s ASSURED criteria (affordable, sensitive, specific, user-friendly, rapid and robust, equipment-free, and deliverable), a paper-based assay that interfaces with a smartphone application for the quantification of iron fortificants is presented. The assay is based on the Ferrozine colorimetric method. The reaction started after deposition of the 5 µL aqueous sample and drying. After developing color, pixel intensity values were obtained using a smartphone camera and image processing software or a mobile application, Nu3px. From these values, the actual iron concentration from ferrous sulfate and ferrous fumarate was calculated. The limits of detection, quantification, linearity, range, and errors (systematic and random) were ascertained. The paper-based values from real samples (wheat flour, nixtamalized corn flour, and infant formula) were compared against atomic emission spectroscopy. The comparison of several concentrations of atomic iron between the spectrophotometric and paper-based assays showed a strong positive linear correlation (y = 47.01x + 126.18; *R*^2^ = 0.9932). The dynamic range (5.0–100 µg/mL) and limit of detection (3.691 µg/mL) of the paper-based assay are relevant for fortified food matrices. Random and systematic errors were 15.9% and + 8.65 µg/g food, respectively. The concept can be applied to limited-resource settings to measure iron in fortified foods.

## 1. Introduction

In recent years, sensor technologies detecting micronutrients (e.g., vitamins and minerals such as iron and vitamin A) have been at the forefront of technology development. These efforts have largely focused on the development of diagnostic tools for assessment of status or deficiency biomarkers in biological samples [1,2,3,4,5]. Few of these sensors, however, are designed to detect micronutrients in food matrices and only very few of those available reach proof-of-concept. In the case of iron, sensing technologies for iron fortificants in food matrices are pertinent to the quality control and compliance monitoring steps of food fortification programs [6]. As large quantities of iron have the potential to cause harm in humans, particularly in malaria-endemic regions, successful and sustainable fortification programs rely on monitoring and evaluation to ensure adequate levels are reached to maximize benefits while reducing any harm [6]. Fortification programs represent the most frequently used and cost-effective nutrition specific intervention for combating iron deficiency anemia (IDA) in vulnerable populations worldwide [7]. There is a limited number of commercially available sensors capable of detecting iron in foods within resource-limited settings, leaving atomic spectroscopy as the most accurate and reliable, but costly, option for monitoring and evaluation of food fortification programs [8].

The characteristics required for a sensor technology implemented in low- and middle-income countries (LMICs) are outlined by adhering to the World Health Organization’s ASSURED (affordable, specific, sensitive, user-friendly, rapid and robust, equipment-free, and deliverable) guidelines for diagnostic technology in LMICs [9,10].

The accepted gold standard method for measuring iron in food samples is through atomic spectroscopy [11]. However, this method of analysis requires major capital expense, trained personnel to operate, and a laboratory often found in universities, private industries, or government agencies. The Ferrozine method for iron determination, however, is relatively inexpensive and simpler than atomic spectroscopy and has been applied for iron determination in solutions [12,13]. This method involves the formation of a Fe(Sodium 4-[3-pyridin-2-yl-5-(4-sulfophenyl)-1,2,4-triazin-6-yl]benzenesulfonate)_3_^2+^ magenta complex in solution, with a pH range of between 4–9, which can then be analyzed by a spectrophotometer at 562 nm [12]. The Ferrozine method has been applied to foods for total iron measurements, such as meats and fortified foods such as yogurts, dry milk, and cereals. However, the use of nitric acid required in the sample preparation, as well as the requirement of a relatively sophisticated spectrophotometer, limits the method’s operational feasibility in LMICs without proper laboratory settings and safety measures in place [14,15].

In the present work, we have developed a novel paper-based variation of the Ferrozine method to determine added iron (i.e., ferrous fumarate, ferrous sulfate) in fortified foods. The method can be particularly useful for government agencies or the food industry to assure the quality of fortified foods entering the market. The method adaptation does not require nitric acid in the sample preparation (i.e., measures unbound, added iron that’s readily soluble in water or dilute acid), increases the working range three-fold, is relevant for the range of iron used in fortification programs, and is potentially inexpensive. The transducer used is a smartphone camera, eliminating the need for sophisticated laboratory equipment, such as a spectrophotometer. After taking a photo, the iron concentration can be determined using an open-access image analysis software or a novel mobile app, Nu3px. The iron paper-based assay is an improvement to previous assays by requiring fewer capital costs, trained personnel, expensive reagents, and sample preparation. The present method has been successfully applied to commonly fortified samples, including wheat flour (Tanzania), infant formula, and nixtamalized corn flour (Mexico). Its analytical figures of merit are relevant for the context of analyzing iron in fortified foods and its applicability to LMICs follows the WHO’s ASSURED guidelines.

## 2. Materials and Methods

### 2.1. Materials and Instrumentation

The following materials were purchased from Sigma Aldrich (St. Louis, MO, USA): Ferrozine (3-(2-Pyridyl)-5,6-diphenyl-1,2,4-triazine-p,p′-disulfonic acid monosodium salt hydrate, C_20_H_13_N_4_NaO_6_S_2_·xH_2_O), ammonium hydroxide solution (28% NH_3_ in H_2_O, ≥ 99.99%), Whatman^®^ No.4, and Whatman^®^ 1PS paper. The following materials were purchased from Fisher Chemical (Waltham, MA): Ammonium acetate (C_2_H_7_NO_2_) and 70% nitric acid (HNO_3_). The following materials were purchased from Spectrum Chemical (New Brunswick, NJ, USA): Ferrous fumarate and hydroxylamine hydrochloride (NH_2_OH·HCl). Hydrochloric acid (5 N) was purchased from Ricca Chemical Company (Arlington, TX, USA). Sodium iron ethylenediaminetetraacetate (Ferrazone XF) was purchased from Akzo Nobel (Amsterdam, Netherlands). TraceCERT standard stock solutions (1000 µg/mL) and TraceCERT multielement standard solution 6 for ICP was purchased from Fluka Analytical (Waltham, MA, USA). Reference standards for corn meal and wheat flour were purchased from High-Purity Standards (Charleston, SC, USA).

Organic infant formula (Earth’s Best; Boulder, CO, USA) and golden corn masa flour (Bob’s Red Mill; Milwaukie, OR, USA) were purchased from Amazon. Fortified wheat baking flour (Azam) was purchased at a market in Arusha, Tanzania, and transported back to University of Illinois at Urbana-Champaign for subsequent analysis. Raw yellow corn (variety 845) was collected from Oaxaca, Mexico.

For atomic emission spectroscopy (AES), the 4100 MP-AES, equipped with an SPS 3 sampler (Agilent Technologies, Santa Clara, CA, USA), was used. In vitro spectrophotometry was conducted using a Genesys 10S UV-Vis Spectrophotometer (Thermo Fisher Scientific, Waltham, MA, USA). Photos were taken using an iPhone 8 iOS 11 and a prototype polylactic acid 3D printed (Ultimaker 2+) photo box attachment (see Figure S1) and subsequent image analysis was conducted using Fiji image analysis software [16].

### 2.2. Preparation of Solutions and Samples

Concentrations and proportions of color-changing reagents were used as described by Viollier et al., with slight modifications for enhanced reaction on paper [13]. A total of 1.4 M hydroxylamine hydrochloride (reagent A) was prepared in 2 M hydrochloric acid. A total of 0.1 M Ferrozine (reagent B) was prepared in 10^−1^ M ammonium acetate. A total of 10 M ammonium acetate (reagent C) was adjusted to pH 9.5 with ammonium hydroxide.

Iron standard solutions were prepared by making serial dilutions from 1000 µg/mL standard solution in deionized water to the following concentrations: 100, 50, 25, 10, 5, 2.5, 1.0, and 0.5 µg/mL. Fortified corn flour samples were prepared by adding and mixing ferrous fumarate for 5 min. High, medium, and low concentrations of fortificants in food samples followed WHO’s recommendations for iron fortification per country consumption rate (60, 30, 20 ppm, respectively) [17].

Nixtamalized corn flour, to evaluate the Nu3px app using corn from Mexico, was made by boiling 5 kg of Oaxaca 845 corn in 15 L of water and 50 g of Ca(OH)_2_ and leaving it overnight for 18 h. Nixtamalized corn was ground to masa in a pilot milling and dried to flour using a flash drier (250 °C).

Fortified food samples (2.5 g) were weighed in 50 mL centrifuge tubes before the addition of 10 mL of 0.25 M HCl solution. Contents were vigorously shaken for 10 s and let to settle for 30 min to allow most of the particulate matter to precipitate to the bottom, separated from an almost clear supernatant.

### 2.3. Iron Analysis

In vitro spectrometry analysis of iron using the Ferrozine method was conducted as outlined by Viollier et al. [13]. Iron analysis using atomic emission spectroscopy (AES) was conducted following the procedure from the AOAC Official Method 984.27 [18].

### 2.4. Fabrication of Paper-Based Assay

**Figure 1:**
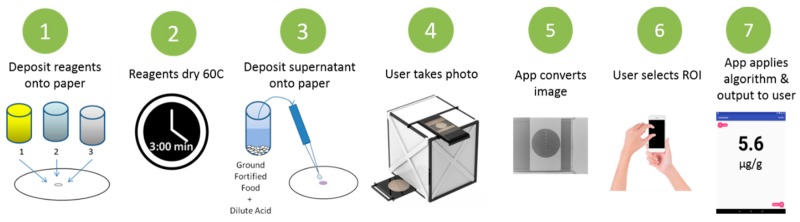
Schematic for the recommended procedure for preparing the paper-based assay, sample deposition, and analysis.

To prepare the paper-based substrate, 3 µL of reagent A, 2 µL of reagent B, and 2 µL of reagent C were subsequently deposited onto Whatman 1PS paper using a 2–20 µL pipette. In between each addition of reagent, the paper was placed in an incubator oven at 60 °C (Heratherm, Thermo-Fisher Sci., Waltham, MA, USA) for 3 min (Figure 1). This paper is now ready for sample detection.

### 2.5. Paper-Based Measurement Procedure

An aliquot (5 µL) of the food sample supernatant or standard solution was deposited onto the detection zone (i.e., containing the dried reactants) of the Whatman 1PS paper using a 2–20 µL pipette. The paper turns magenta as soon as the iron reacts with the Ferrozine reagent. To dry the reactants, the paper was placed in the incubator oven at 60 °C for 3 min. The paper can also be dried by leaving the strips at room temperature for 30 min. The color reaction does not require temperature and it is not affected by it. After the stable formation of a dry magenta spot on the paper, the paper and a standard curve were placed in the 3D-printed box before the photo was taken using an iPhone 8 camera. Including the standard curve with the test image increases the reliability of mean pixel intensity by providing a reference for the camera to adjust its pixels. The image was subsequently analyzed using the Fiji software [16] on a Dell PC desktop computer. Imaging analysis included conversion of the image (magenta spot) to 8-bit grayscale, color inversion, and determination of the mean pixel intensity of the detection zone. The mean pixel values were plotted against the log concentration of iron to create calibration curves and a linear regression equation was used to determine the iron concentrations in food samples. Converting the analyte concentrations to the log scale is a common method deployed for linearizing calibration curves for paper-based assays [19]. Its appropriate use and best fit are described later.

### 2.6. Interference Studies

Several potentially interfering minerals (Zn, K, Ca, Na, Cu, Se, B, Mn, P, Mg, Mo, Co) were individually tested (at 1000 µg/mL) using the Ferrozine assay in solution and compared to the Ferrozine assay with water as a control (see Figure S2). Elements that produced a visible color change (Zn, Se, Cu, Co) from the control solution were tested further to quantify interference, using methodology from Westgard [20]. Fortified corn starch (*n* = 5 replicates per interferent) was tested using the above paper-based procedure, containing 40 µg Fe/g starch in the presence of minerals added in the amounts often found in corn flour (6.3 µg Zn/g flour [21], 0.164 µg Se/g flour [22], 1.20 µg Cu/g flour [23], 10.3 µg Co/g flour, determined by AES). Differences between the average of iron fortified corn starch without interferent (*n* = 5) and the average of iron-fortified corn flour with interferent (*n* = 5) were calculated to find the average systematic error (bias) due to each interferent.

### 2.7. Recovery and Spike Studies

Recovery studies in dilute acid solution (*n* = 3) were conducted using ferrous fumarate at 2 spiked levels (10 and 20 µg/mL). Recovery studies (*n* = 4) were conducted at 3 spiked fortification levels (12, 36, and 60 mg Fe/kg) in fortified corn flour (ferrous fumarate, 37.92 mg Fe/kg). Apparent recovery % ± %CV was calculated. A spike study was performed in an aqueous solution in the presence of potentially interfering compounds (50 µg/mL) and spiked with 50 µg/mL of atomic iron. Trials (*n* = 8) were conducted in paper and measured with the above recommendation.

### 2.8. Calculations and Statistical Analysis

The limit of detection was determined by calculating the output of the blank (reagents only) + 3σ of the blank (*n* = 20) [20]. The working range was determined by the range in which a reliable signal (*n* = 5) is produced linearly against log iron concentration (*R*^2^ > 0.95). Sensitivity was calculated by (Δ output response/Δ concentration) of the working range values. A method comparison plot was constructed by plotting the output of the paper-based method vs. the AES method, and the Pearson coefficient (*r*) was determined. Means, standard deviations (SD), calculated outputs, *R*^2^ values, coefficients of variation (%CV = (SD/mean) × 100)), and standard curve and method comparison plots were calculated and constructed using Microsoft Excel. A regression standardized residual plot was constructed and the correlation coefficient, bivariate correlation, and paired *t*-test (confidence interval 95%) were calculated on IBM SPSS Statistics 24 [24]. Systematic error (bias) was determined by taking the mean ± standard deviation of the differences between the gold standard method and the paper-based method [20]. The random error was determined by calculating the %CV of the tests’ within-day trial [20]. The apparent recovery (R_A_) equation (Equation (1)) by Burns et al. were used to determine the % recovery from spiked assays [25].

(1) $$R_{A}=\frac{x_{A}\left(O+S\right)-x_{A}\left(O\right)}{x_{A}\left(S\right)},$$

where x_A_(O+S) refers to the original + spiked output values, x_A_(O) refers to the original sample output value, and x_A_(S) refers to the output value of the spiked sample. All analyses are derived from an analytical procedure by a calibration curve.

### 2.9. Development and Evaluation of Mobile Application

A user-friendly mobile application (app) was developed for Android. The app, called Nu3px, allows the end-user to take a photo with the light-tight box directly within the app. Then, upon accepting the photo, the app processes the image (8-bit grey scale, inversion) and allows the user to select the region of interest for the mean pixel measurement. A pilot study testing the app’s feasibility for implementation was carried out in Querétaro, Mexico using corn flour samples collected in Oaxaca and spiked with ferrous sulfate (5 fortification levels, *n* = 4 replicates, 3 measurements). A method comparison plot and the bivariate correlation statistics were obtained to compare the Nu3px vs. the Fiji PC software analyses.

## 3. Results

### 3.1. Iron Measurements of Standard Solutions

**Figure 2:**
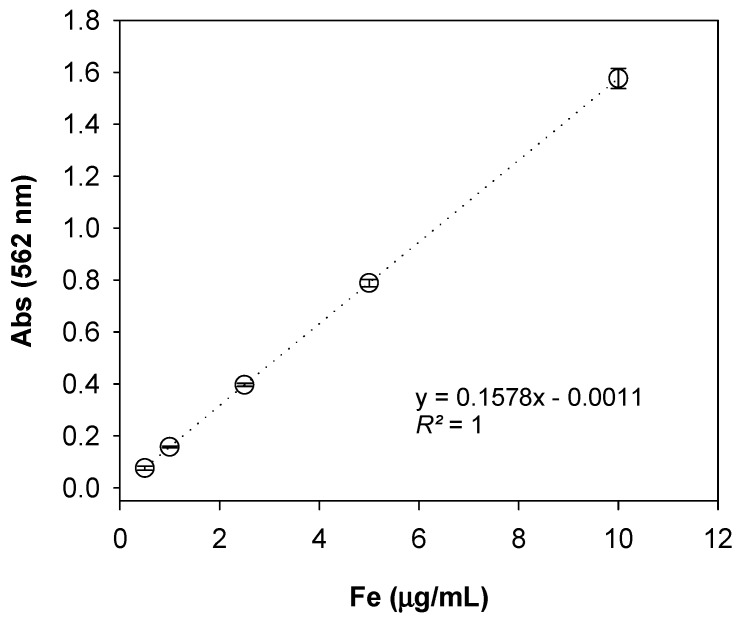
Standard calibration curve of the conventional Ferrozine assay in vitro. Absorbance was measured at 562 nm for varying levels of atomic Fe concentration (*n* = 5) and the means ± SD are shown.

Calibration curves were constructed by analyzing iron stock solutions (0.0, 0.5, 1.0, 2.5, 5.0, 10.0, 25.0, 50.0, and 100.0 µg Fe/mL; *n* = 5). The in vitro method calibration curve’s maximum was 10.0 µg Fe/mL, as the intensity reached too high to read in the spectrophotometer beyond 10.0 µg Fe/mL (Figure 2).

**Figure 3:**
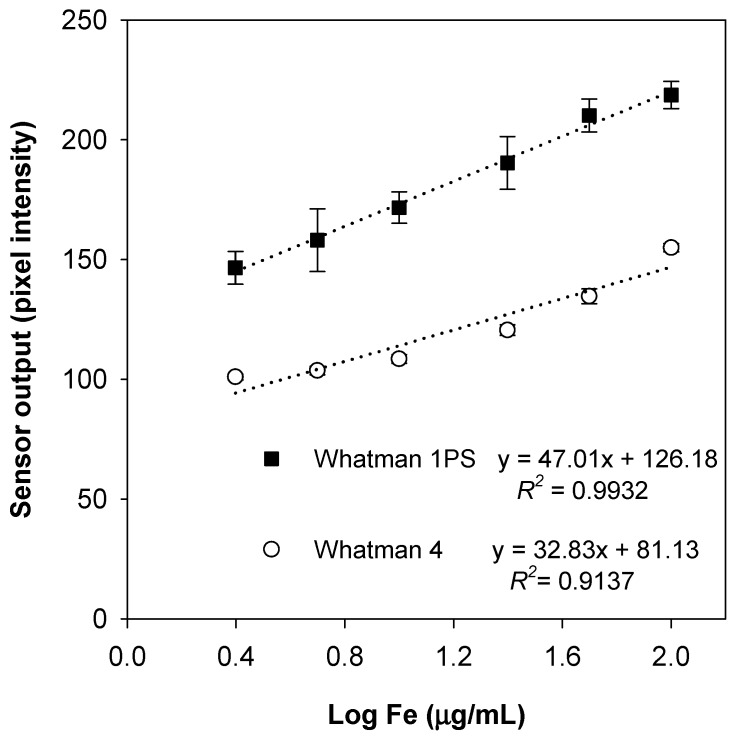
Iron standard curves using two paper platforms. Data points represent pixel characterization (Mean ± SD) as a response to iron concentrations (6 fortification levels, *n* = 5 of each) using Whatman 1PS and Whatman no. 4 papers.

Previously reported paper-based assays have used either platform, but the hydrophobicity of the Whatman 1PS papers have shown an increase in sensitive outputs for other assays [26,27]. In the current study, Whatman 1PS papers showed an advantage by an increase of 43% in sensitivity and an improvement in the linearity (*R*^2^ values) of concentrations up to 100 µg/mL (Figure 3) and, thus, were used hereafter for other determinations. The linear regression curve found using Whatman 1PS paper (y = 47.01x + 126.18) was used to plot a regression standardized residual curve to evaluate the fitted model used in all subsequent experiments and was found to have a random pattern. The determination coefficient was 0.9932 (*p* < 0.01), indicating an almost perfect dose response relationship [28]. The use of correlation coefficients, as well as evaluating the residual plot, has been recommended in the development of paper-based assays [19].

### 3.2. Analytical Figures of Merit

#### 3.2.1. Range

The working range of the paper-based assay was determined to be 5.0–100 µg Fe/mL and is the range in which a reliable signal is produced in a positively linear range. In order to obtain a positive, linear equation, the photo must be inverted for a positive pixel intensity relationship and the sensor output must be compared to the log Fe concentration. This range was found to be superior to the Ferrozine method (0–10.0 µg Fe/mL) for its application to fortified foods, which require sensitivities in the order of 10–25 µg Fe/mL, without additional dilutions. These dilutions represent additional steps and costs in determinations.

#### 3.2.2. Sensitivity

The limit of detection (LOD) for the paper-based assay was determined to be 3.691 µg Fe/mL (*n* = 20), at which point the sensor output is reliably detectable above the background noise. This LOD is low enough to be a relevant assay for fortified foods (i.e., 10–25 µg Fe/mL without dilution). The sensitivity of the paper-based assay within the working range was determined to be 0.7396.

#### 3.2.3. Specificity

Table 1 reports results from the interference studies (*n* = 5). In the presence of commonly found amounts of Zn, Se, Cu, and Co, using corn starch as a sample matrix, mean deviation (systematic error or bias due to interferents) from the expected Fe amount was found to be +1.01 µg/g. Thus, some amount of interference at naturally occurring levels of these minerals of interest in corn flour can be assumed. Similar findings for copper and cobalt as interferents of the Ferrozine assay have been reported elsewhere [12].

### 3.3. Iron Determination in Foods

**Figure 4:**
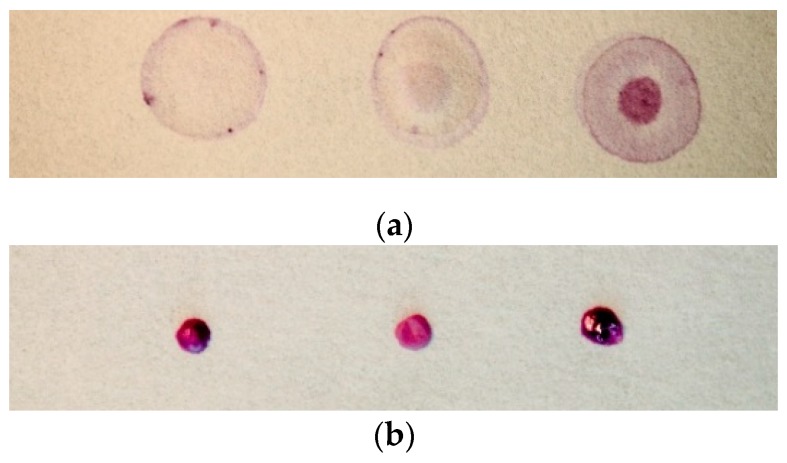
Comparison of Whatman papers. Fortified food matrices (from left to right, corn flour, 10 µg/mL NaFeEDTA, Tanzanian wheat flour, 8.3 µg/mL Fe type unknown, infant formula, 12 µg/mL FeSO4). Whatman no.4 papers (**a**) resulted in heterogeneous and inconsistent output. Using the same procedure, Whatman 1PS papers (**b**) showed improved sensitivity and reliability with food matrices.

Iron in foods was determined using both the AES and paper-based methods. The AES was calibrated with serially diluted stock solutions of iron and the results were adjusted by comparing NIST wheat flour and corn meal standards for internal reference. When measuring food matrices, Whatman no. 4 papers produced heterogeneous and inconsistent results (Figure 4a). Heterogeneous color change is a common issue in colorimetric paper-based assays [19]. These are due to inconsistent dispersion of reactants on the paper and capillarity due to the compatibility of the paper material and the aqueous samples. This issue was overcome after selecting the Whatman 1PS paper. This platform showed no color dilution or any other potential food matrix interference due to its hydrophobic nature. This allowed the supernatants to lay on top of the paper with little heterogeneous reaction and color dispersion (Figure 4b).

### 3.4. Accuracy and Determination of Systematic Error

**Figure 5:**
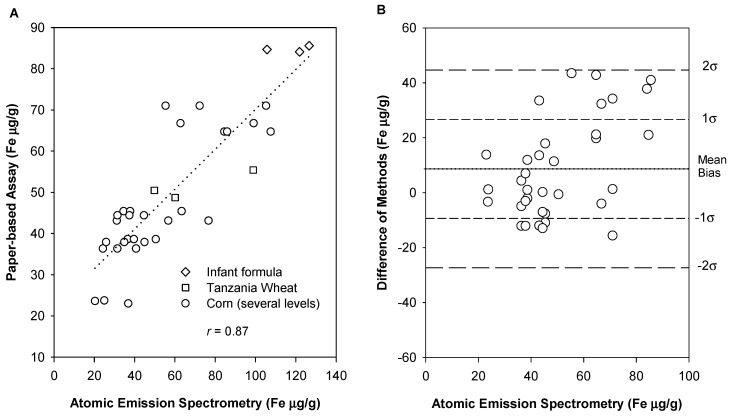
(**A**) Method comparison plot between the gold standard method and the paper-based assay for samples low (*n* = 9), medium (*n* = 9), and high (*n* = 8) in ferrous fumarate fortified corn flour, Tanzanian wheat flour (*n* = 3), infant formula (*n* = 3), and unfortified corn flour (*n* = 3). The Pearson coefficient (*r* = 0.87) indicates a high positive linear relationship [28]. (**B**) Bland–Altman plot. All but 1 data point are within 2σ and the majority of data points (71%) are within 1 standard deviation.

A total of *n* = 35 food samples (*n* = 26 corn flour fortified with ferrous fumarate, *n* = 3 unfortified corn flour, *n* = 3 infant formula fortified with ferrous sulfate, and *n* = 3 fortified wheat flour collected in Tanzania) were analyzed by both methods (Figure 5). The atomic emission spectroscopy method was found to have a %CV of 4.24. The Pearson correlation coefficient from the method comparison plot (*n* = 35) was 0.865, indicating a high positive linear relationship [28]. Westgard et al. argues that when the Pearson coefficient from the method comparison plot is less than 0.99, the method bias (mean of the differences) is an appropriate measure of systematic error [20]. As such, the bias and systematic error of the assay is +8.65 µg/g ± 18.00 µg/g. These data points represent assays that have not been diluted beyond the 2.5 g sample in 10 mL 0.25 M HCl. The Bland–Altman plot shows more variability at higher concentrations. This is likely due to the logarithmic calibration curve and this source of variation is expected to be diminished, for example, by using a higher initial dilution.

Recovery assays were conducted and % apparent recoveries [25] were calculated. Recovery experiments are used in preliminary method development to determine proportional error [20]. Dilute acid solutions (without matrix) were spiked to 10 and 20 µg/mL Fe using ferrous fumarate. Percent mean apparent recoveries ± CV% (*n* = 3) were 118.3 ± 5.8 and 108.3 ± 9.4, respectively, indicating that all of the iron from ferrous fumarate was dissolved using the currently indicated extraction procedure. To test recovery with a matrix, fortified corn flour with ferrous fumarate (37.92 mg Fe/kg) were spiked with 12, 36, and 60 mg Fe/kg using atomic iron standard solutions. Percent mean apparent recoveries ± CV% (*n* = 4) were calculated to be 101.4 ± 22.9, 126.6 ± 16.7, and 118.2 ± 9.8, respectively. This supports the previous findings that the paper-based sensor has a positive bias, especially at higher concentrations.

A spike study (see Table S1) was performed in a mineral solution in the presence of the following potentially interfering compounds: Al, Sb, Ba, Pb, B, Ca, Cd, Cr, Co, K, Cu, Li, Mg, Mn, Mo, Na, Ni, P, Si, Ti, V, and Zn at 50 µg/mL. Iron was spiked from 50 to 100 µg/mL. The deviation (*n* = 8 trials) was found to be + 5.72 ± 7.2%. This can be explained by the cobalt interference.

### 3.5. Precision and Determination of Random Error

In order to determine preliminary precision and, moreover, the random error of the assay, a within-day replication test, as outlined by Westgard [20], was conducted. One sample of fortified corn flour (36.35 mg Fe/kg flour) was tested *n* = 9 times to determine precision within 1 day. The random error (CV%) was 15.9%. Results from a paired *t*-test (confidence interval 95%) showed that, in corn flour with an Fe concentration ranging 20–60 mg Fe/kg flour, iron concentrations obtained from either method (i.e., AES vs. paper-based) were the same (*p* > 0.05).

### 3.6. Pilot Test with Nu3px Mobile App

**Figure 6:**
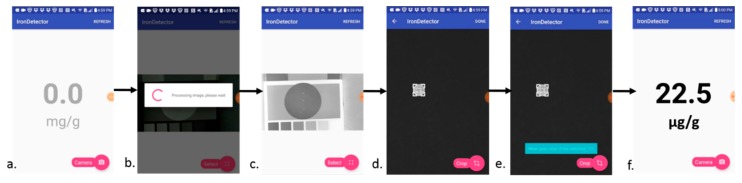
Screenshots of the Nu3px app analysis flowchart. (**a**). Home screen upon opening app. (**b**,**c**). After the user takes a photo within the app, the app automatically processes the image (i.e., converts image to 8-bit grey scale, invert). (**d**) The user selects the detection zone using a pinch and drag circle focus. (**e**) The app measures the mean grey pixel value. Steps (**d**,**e**) can be repeated for replicates. (**f**) Once the user has measured all the replicates, the app applies the calibration curve algorithm and averages the result between the x number of replicates for a final output in the desired concentration correcting for dilution.

**Figure 7:**
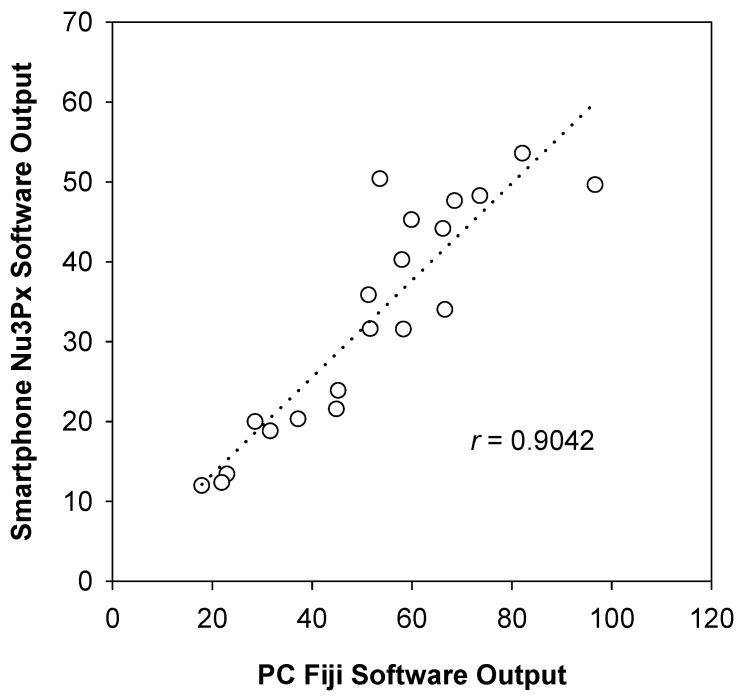
Method comparison plot of several samples of corn spiked with ferrous fumarate analyzed via two methods, as follows: The computer-based Fiji software and the smartphone-based Nu3px app. Data are means of five spike levels (µg Fe/g flour) measured four times with either software analysis tool.

The Nu3px app, version 1.0, was developed to perform the same image processing functions as Fiji image processing software (Figure 6). In order to test feasible implementation in Mexico as a case study, the proposed mobile app coupled with the paper-based assay was tested on corn samples obtained in Querétaro, Mexico. Samples of fortified nixtamalized yellow corn flour were measured on paper and performed using both the app and the Fiji software. A bivariate correlation analysis showed a statistically significant correlation (*p* < 0.01) and a Pearson correlation coefficient of 0.904 (Figure 7), indicating a strong positive relationship.

As the Fiji’s image analysis functions used (convert to 8-bit grey scale, invert) are not open access, the functions to do similar operations in Nu3px app are slightly different. This difference in image conversion can be seen by the variability in the method comparison plot (Figure 7). This pilot test demonstrates the interchangeability of the Fiji software with the app and the feasibility for its implementation in Mexico.

## 4. Discussion

Mass food fortification is a common strategy used by many countries to address specific micronutrient gaps prevalent in their populations [6]. Despite supportive legislation, most fortification programs in low-resource settings lack the access to sophisticated equipment to ascertain product quality. This includes the determination of the amount of added nutrients in the final food product and its variability from batch to batch. Importantly, in low-income settings, government agencies responsible for monitoring fortification programs lack the ability to adequately monitor the food supply. Thus, low-cost sensing tools that can be used by both governments and the food industry could prove useful in providing accurate and reliable data to support fortification programs.

Paper-based assays are often the preferred methods of analysis due to their low-cost, ease of use and interpretation, and portable nature [19]. Recent paper-based assays designed for food matrices have focused on detection and quantification of food additives (i.e., food colorings), pathogens, pesticides, herbicides, and toxic trace metals [29]. The same efforts in design and development of ASSURED-based assays can be used to address current limitations in micronutrient analysis in fortified foods.

To the best of the authors’ knowledge, a paper-based sensor for iron determination in solid foods currently does not exist, likely due to the decrease in reliability with a bulky food matrix. It is estimated that industrially processed fortified foods, such as cereal flours, will have an intrinsic 15% variability in their content of iron and other minerals, depending on the amount of micronutrients added due to particle size and the characteristics of the food matrix [30]. Similarly, the CV% of precision due to random error was found to be 15.9% with the current method of analysis. The present study overcame additional challenges of imprecision partly by employing a colorimetric reaction on Whatman 1PS paper for a simple ASSURED-designed paper-based assay.

Though other colorimetric iron assays are possible on paper [31], the Ferrozine assay was utilized in the present work based on its versatility in terms of temperature, humidity, and pH range [12]. This allows the current method to be modified easily in the future to measure other food matrices under various environments.

### 4.1. Comparison to Other Methods

No other paper-based assay exists for ground fortified foods. As such, the novelty of the assay lies within this unique aspect and is a promising discovery for modifying other colorimetric methods of analysis to paper. The assay’s figures of interest are comparable or improved over to other Fe determination assays including the limit of detection, the working range, the equipment needed, time, and the estimated cost of materials (Table 2). The cost of materials was estimated via online quotes for materials and adjusted for the quantity of materials used in each assay. For the last two methods, the fabrication of the strip sensor required more sophisticated steps and was not calculated and the cost of the atomic emission spectroscopy was quoted by laboratories at the University of Illinois at Urbana-Champaign. This latter cost includes labor. Similar to other methods [32,33,34], this paper-based assay can be used as a qualitative “naked eye” determination of iron. For this, a simple magenta intensity palette can be designed to match specific iron concentrations qualified by the dilution used. As shown, the addition of the smartphone and the Nu3px app facilitates the actual estimation of iron concentration directly [35,36]. The use of smartphones for sensing applications has been reported steadily in literature [37]. Similarly, the ownership of smartphones is increasing in low-income settings [38]. For these reasons, the authors argue that this is a viable method for the detection of iron in fortified foods and deserves confirmation via field validation studies.

### 4.2. Limitations and Future Studies

While this work presents a novel adaptation of a method for the determination of iron in foods that fits the WHO’s ASSURED conceptual framework for diagnostic technology in low-resource settings, it is not a suggested replacement for the gold standard methods until a thorough field-validation study has been conducted in one of the LMICs. Optimization of the sampling paradigm is necessary. For example, the assay used tools that might not be feasible in LMICs, such as an analytical balance for sample weight, a volumetric pipette for measuring sample acid dilution, and a micropipette for sample deposition. The authors suggest that future studies focus on collecting data with simplified and cost-effective tools to determine its effectiveness in LMICs. Other limitations are associated with the current assay’s systematic and random errors. This assay was developed for readily soluble forms of iron in dilute acid (i.e., ferrous fumarate and ferrous sulfate), as there was a higher variability in the recovery assays when using ferric pyrophosphate in the current extraction procedure. It is possible that a stronger acid and a longer extraction time would be required for complete dilution of these iron forms. Other common iron fortificants (e.g., electrolytic iron) will need to be evaluated under the current extraction procedure in future studies. In order to ensure that a reliable and accurate output can be measured without positive bias, future studies should include the incorporation of a calibration mechanism, which will help correct the positive bias, as it was the case in these studies. In addition, a larger sample size (*n* > 40) should be used to fully validate the sensor and correct for any potential systematic error. If the bias is associated with higher concentrations, a way to address this is by diluting the sample. Upon completion of these future studies, the present method can be considered as an ASSURED alternative to the current expensive analytical methods for iron determination in fortified foods.

## 5. Conclusions

The present work presents an adaptation of the original Ferrozine assay to a paper-based platform for the determination of iron in commonly fortified foods, such as wheat, nixtamalized corn flour, and infant formula. The paper-based assay showed dependable accuracy, reliability, specificity, and sensitivity for the range of iron found in these foods. The method, including the smartphone app, was pilot tested in Mexico. The method aligns well with the WHO’s ASSURED guidelines for diagnostic technology in resource-limited settings and, thus, can be implemented in the quality control or compliance monitoring steps of countrywide mandatory or voluntary food fortification programs, such as those in LMICs. Optimization is necessary to improve these performance indicators. Future studies will include a thorough validation study of samples collected in LMICs, applying the new smartphone app, which is a user-friendly interface for low-literacy populations.

## Figures and Tables

**Table 1:** Interference Studies.

Interferent (µg/g)	Mean Measurement ± SD (µg Fe/g)	Difference (µg Fe/g)
Unfortified (0)	0.95 ± 0.23	N/A
Fe (40) + no interferent	48.45 ± 1.60	N/A
Fe (40) + Zn (7.0)	44.34 ± 5.73	−4.11
Fe (40) + Se (1.0)	57.19 ± 6.42	8.74
Fe (40) + Cu (2.0)	49.28 ± 5.13	0.83
Fe (40) + Co (11)	47.02 ± 8.35	−1.43
	Avg. Interference	1.01

Selected minerals were added to fortified corn starch (40 µg Fe/g starch) at naturally occurring amounts (*n* = 5). Corn starch, both with and without interferents, was measured and their average differences were calculated to estimate systematic error due to interferents. [20] Co and Cu are expected to have interferences in the presence of 8:1 and 2:1 Co and Cu concentration, respectively, as found by Stookey [12].

**Table 2:** Comparison of iron determination methods.

Sensor	Matrix	LOD	Working Range	Equipment Needed	Time	Estimated Cost of Materials (USD)
Current work	Fortified foods dispersed in diluted acid	3.691 µg/mL or 18.5 ng (dried)	5.0–100 µg/mL	Reactive paper + Smartphone + Light-tight box	5 min	$0.29
Original in vitro Ferrozine assay [13]	Several matrices dispersed in diluted acid	0.5 µg/mL	0.5–10 µg/mL	Liquid reagents + Spectro-photometer	20 min	$3.55
Trace metal paper-based sensor [31]	Metal containing aerosols	1500 ng	1.5–10 µg	Strip + Image Scanner	Sample collected over 8-h	$0.013 (estimated by authors)
Visual strip sensor [39]	Ground water and fruit juices	0.02 µg/mL	0.02–2.0 µg/mL	Visual Strip + Spectrophotometer	15 min	N/A
Atomic emission spectroscopy [40]	Any	0.5 µg/mL	0.5–4.0 µg/mL	Flame Atomic Absorption Spectrometer	1 h 30 min	$25

The present method is presented against three other alternative methods to contrast its performance parameters and ASSURED design characteristics.

## Supplementary Materials

The following are available online at https://www.mdpi.com/2072-6643/11/7/1673/s1, Figure S1. Design of 3D-printed photo box with polylactic acid material for iPhone 8, Figure S2. Interference study, Table S1. Spike/recovery study.
