# Supplementary material for: Development of a Paper-Based Sensor Compatible with a Mobile Phone for the Detection of Common Iron Formulas Used in Fortified Foods within Resource-Limited Settings
Source: Nutrients. 2019 Jul 21;11(7):1673. doi: 10.3390/nu11071673 (PMC6682910; doi:10.3390/nu11071673)
Supplement: Supplementary file 1 [file nutrients-11-01673-s001.pdf]

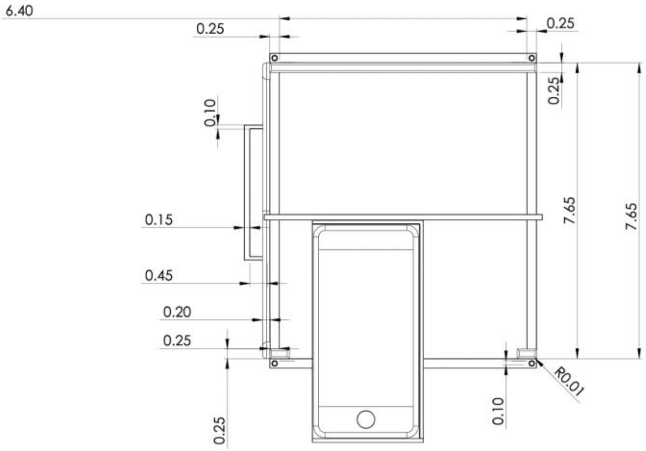

(**A**)

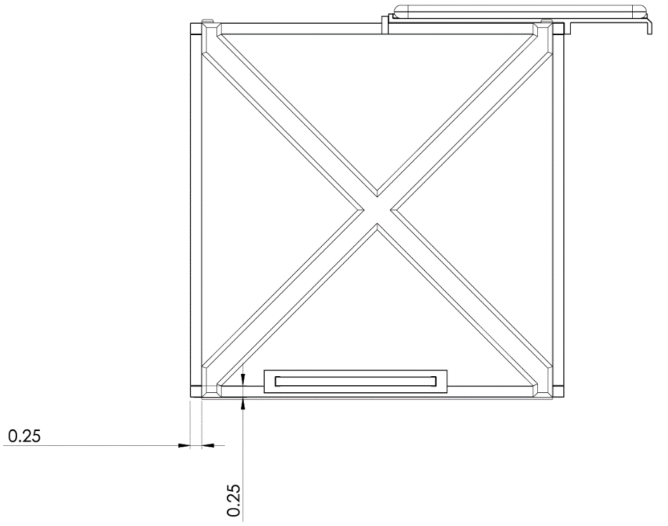

(**B**)

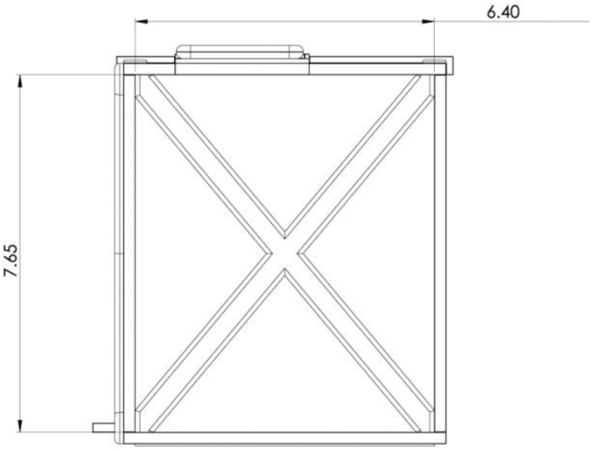

(**C**)

Figure S1. Design of 3D-printed photo box with polylactic acid material for iPhone 8. (**A**) View from the top. (**B**) View from the left side. (**C**) View from the back. Dimensions are all in inches. Box weighed approximately 500 g. Designed with AutoCAD.

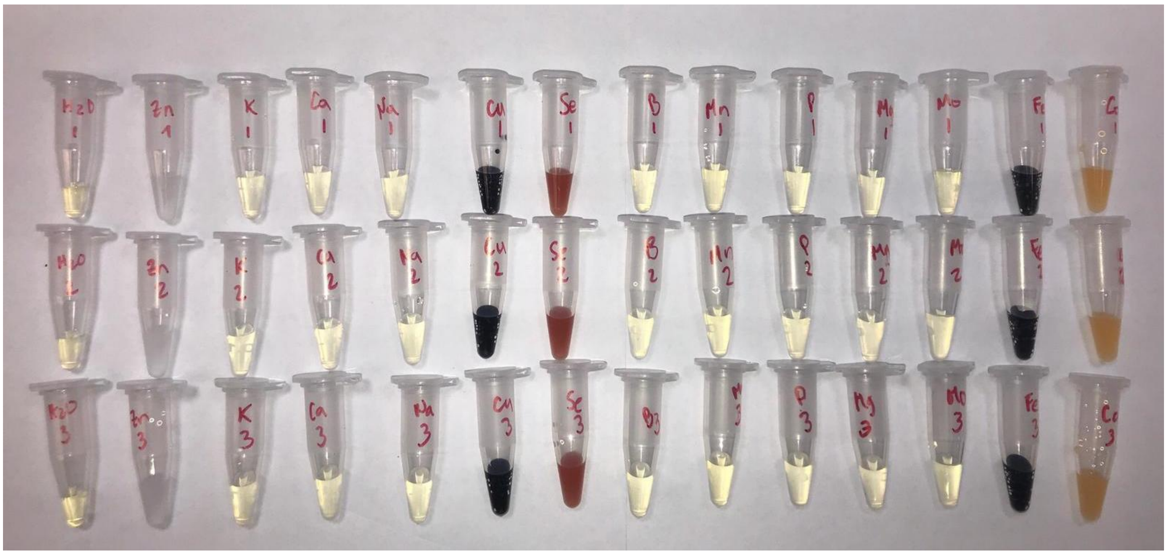

Figure S2. Interference study. Single elements (from left to right: control, Zn, K, Ca, Na, Cu, Se, B, Mn, P, Mg, Mo, Fe, and Co at 1000 μg/mL) were reacted (*n* = 3 replicates) in solution using the original Ferrozine assay and compared to the control (water). Zn, Cu, Se, and Co exhibited a visible response to the naked eye, indicating a possible interference and were tested further. The concentration tested was in excess of what is normally present in fortified food samples. Due to the high concentration, the iron sample (second to last) showed a strong dark response.

Table S1. Spike/recovery study.

Expected Fe (μg/mL)	Measured Fe (μg/mL)	Deviation (%)
100	102.1	2.14
100	109.8	9.76
100	115.2	15.21
100	101.1	1.13
100	101.0	0.98
100	105.5	5.5
100	95.6	−4.38
100	115.4	15.41
Mean ± SD	105.7 ± 7.2	5.72

Iron (50 μg/mL) was spiked to a solution containing potential interferences (50 μg/mL): Fe, Al, Sb, Ba, Pb, B, Ca, Cd, Cr, Co, K, Cu, Li, Mg, Mn, Mo, Na, Ni, P, Si, Ti, V and Zn. The final concentration of the Fe was set at 100 μg/mL.
